# The African swine fever virus I10L protein inhibits the NF-*κ*B signaling pathway by targeting IKK*β*

**DOI:** 10.1128/jvi.00569-23

**Published:** 2023-09-28

**Authors:** Xing Chen, Lian-Feng Li, Zhong-Yuan Yang, Meilin Li, Shuai Fan, Lan-Fang Shi, Zi-Yu Ren, Xue-Jing Cao, Yuhang Zhang, Shichong Han, Bo Wan, Hua-Ji Qiu, Gaiping Zhang, Wen-Rui He

**Affiliations:** 1 International Joint Research Center of National Animal Immunology, College of Veterinary Medicine, Henan Agricultural University, Zhengzhou, Henan, China; 2 State Key Laboratory for Animal Disease Control and Prevention, National African Swine Fever Para-Reference Laboratory, National High-Containment Facilities for Animal Disease Control and Prevention, Harbin Veterinary Research Institute, Chinese Academy of Agricultural Sciences, Harbin, Heilongjiang, China; 3 College of Veterinary Medicine, Henan Agricultural University, Zhengzhou, Henan, China; 4 Longhu Laboratory, Henan Agricultural University, Zhengzhou University, Zhengzhou, China; Lerner Research Institute, Cleveland Clinic, Cleveland, Ohio, USA

**Keywords:** African swine fever virus, inflammatory response, I10L protein, IKK*β*

## Abstract

Proinflammatory factors play important roles in the pathogenesis of African swine fever virus (ASFV), which is the causative agent of African swine fever (ASF), a highly contagious and severe hemorrhagic disease. Efforts in the prevention and treatment of ASF have been severely hindered by knowledge gaps in viral proteins responsible for modulating host antiviral responses. In this study, we identified the I10L protein (pI10L) of ASFV as a potential inhibitor of the TNF-*α*- and IL-1*β*-triggered NF-*κ*B signaling pathway, the most canonical and important part of host inflammatory responses. The ectopically expressed pI10L remarkably suppressed the activation of NF-*κ*B signaling in HEK293T and PK-15 cells. The ASFV mutant lacking the *I10L* gene (ASFV_ΔI10L_) induced higher levels of proinflammatory cytokines production in primary porcine alveolar macrophages (PAMs) compared with its parental ASFV HLJ/2018 strain (ASFV_WT_). Mechanistic studies suggest that pI10L inhibits IKK*β* phosphorylation by reducing the K63-linked ubiquitination of NEMO, which is necessary for the activation of IKK*β*. Morever, pI10L interacts with the kinase domain of IKK*β* through its N-terminus, and consequently blocks the association of IKK*β* with its substrates I*κ*B*α* and p65, leading to reduced phosphorylation. In addition, the nuclear translocation efficiency of p65 was also altered by pI10L. Further biochemical evidence supported that the amino acids 1–102 on pI10L were essential for the pI10L-mediated suppression of the NF-*κ*B signaling pathway. The present study clarifies the immunosuppressive activity of pI10L, and provides novel insights into the understanding of ASFV pathobiology and the development of vaccines against ASF.

**IMPORTANCE:**

African swine fever (ASF), caused by the African swine fever virus (ASFV), is now widespread in many countries and severely affects the commercial rearing of swine. To date, few safe and effective vaccines or antiviral strategies have been marketed due to large gaps in knowledge regarding ASFV pathobiology and immune evasion mechanisms. In this study, we deciphered the important role of the ASFV-encoded I10L protein in the TNF-*α*-/IL-1*β*-triggered NF-*κ*B signaling pathway. This study provides novel insights into the pathogenesis of ASFV and thus contributes to the development of vaccines against ASF.

## INTRODUCTION

African swine fever (ASF) is a highly contagious disease of swine with mortality approaching 100%. Since it was first identified in Kenya in 1921, ASF has currently distributed in 42 countries in five different world regions, with more than 1,120,000 animal losses from January 2021 to March 2023 (1) (https://www.oie.int/en/animal-health-in-the-world/animal-diseases/african-swine-fever /). Although the ASF outbreak has now reached epidemic proportions and has severely affected the global production of swine, the effective vaccines are currently unavailable for the control of ASF except the ASFV-G-ΔI177L vaccine in Vietnam (2).

African swine fever virus (ASFV), the causative agent of ASF, is the only known member of the genus *Asfivirus* within the family *Asfarviridae* (3). ASFV is a multilayered double-stranded DNA (dsDNA) virus that shares structural, genomic, and replicative characteristics with other large nucleocytoplasmic DNA viruses. ASFV encodes more than 160 proteins that enable successful infection, replication, and immune evasion *in vivo* (4 – 6). To date, the functions, subcellular locations, and transcriptional features of only a few viral proteins have been described (6, 7). Large gaps in knowledge regarding the composition of infectious virions, viral proteins responsible for immune evasion, and pathogenesis have severely hindered the development of vaccines (8).

ASFV mainly targets primary macrophages and monocytes (9 – 11), which increase the secretion of the proinflammatory cytokines, such as tumor necrosis factor alpha (TNF-*α*), interleukin-1*β* (IL-1*β*), IL-6, and IL-8 (12). As major components of host antiviral innate immunity, TNF-*α* and IL-1*β* play central roles in controlling the replication of many viruses, such as classical swine fever virus (13) and Japanese encephalitis virus (14). Previous studies have demonstrated that the elevated levels of TNF-*α* play important roles in the pathogenesis of ASF due to its proinflammatory, proapoptotic, and procoagulant profile (15, 16). NF-*κ*B activation is one of the hallmarks following TNF-*α* and IL-1*β* stimulation. Once the plasma membrane-anchored receptors [tumor necrosis factor receptor 1 (TNFR1) or interleukin-1 receptor (IL-1R)] bind to their respective ligands, they recruit and activate adaptors and kinases, including TNFR-associated death domain protein (TRADD), Myeloid differentiation primary response 88 (MyD88), TGF-*β*-activated kinase 1 (TAK1), TAK1-binding protein 2 (TAB2), TAB3, and I*κ*B kinase *β* (IKK*β*) (17, 18). Following the phosphorylation and degradation of I*κ*B*α*, the transcription factor NF-*κ*B is released and translocated to the nucleus, leading to the expression of various cytokines and chemokines and subsequent inflammatory responses (19, 20).

Recently, several ASFV proteins that regulate the activation of NF-*κ*B have been identified. The F317L protein interacts with IKK*β* and suppresses its phosphorylation, which subsequently stabilizes I*κ*B*α* and blocks the activation and nuclear translocation of NF-*κ*B, which results in decreased expression of various proinflammatory cytokines and an increased viral replication efficiency (21). The MGF505-7R protein (pMGF505-7R) inhibits NF-*κ*B activation by binding to IKK*α*, leading to a reduced IL-1*β* production. ASFV lacking the *MGF505-7R* gene has shown reduced virulence in piglets (22). Deletion of the *H240R* gene decreases infectious viral progeny production due to the aberrant virion morphogenesis and enhanced inflammatory cytokine expression, both *in vitro* and *in vivo* (23, 24). The MGF360-12L protein significantly inhibits the host mRNA transcription and the promoter activity of IFN-*β* and NF-*κ*B, as well as the nuclear localization of p50 and p65 by competitively inhibiting the interaction between NF-*κ*B and nuclear transport proteins (25). The early expressed L83L protein (pL83L) specifically associates with IL-1*β*; however, deletion of the *L83L* gene only slightly affects the virulence of ASFV (26). Further in-depth studies on the mechanisms underlying the regulation of inflammatory responses by ASFV are urgently needed and may be helpful in understanding this devastating disease.

In the present study, we identified the ASFV I10L protein (pI10L) as a potential inhibitor of the TNF-*α*- and IL-1*β*-triggered NF-*κ*B signaling pathway. The pI10L contains approximately 170 amino acids (aa), and belongs to the ASFV p22 family (27). Similar to the inner envelope structural protein p22, pI10L has a transmembrane domain at its N terminus (28). However, little is known about the biological function of pI10L (29). Our results demonstrate that pI10L significantly inhibits the TNF-*α*- and IL-1*β*-induced transcription of proinflammatory genes and activation of the NF-*κ*B promoter. The ASFV mutant lacking the *I10L* gene (ASFV_ΔI10L_) induced much higher levels of *CCL2*, *IL-8*, and *TNF-α* than did the parental ASFV HLJ/2018 strain (ASFV_WT_). The phosphorylation of IKK*β*, I*κ*B*α*, and p65 in the ASFV_ΔI10L_-infected primary porcine alveolar macrophages (PAMs) was significantly increased. Mechanistic studies suggest that the K63-linked ubiquitination of the NF-*κ*B essential modulator (NEMO) is dramatically reduced by pI10L, followed by the inhibition of the phosphorylation of IKK*β*. Besides, pI10L inhibits the association of IKK*β* with I*κ*B*α* and p65, which in turn inhibits the phosphorylation and degradation of I*κ*B*α*, as well as the phosphorylation and nuclear translocation of p65, leading to reduced expression of proinflammatory cytokines. Taken together, our findings reveal the immunomodulatory activity of pI10L, which will help better illustrate the immune evasion mechanisms and pathogenesis of ASFV. These efforts will contribute to the development of vaccines against ASF as well as therapeutics to treat the disease.

## RESULTS

### The ASFV pI10L inhibits the TNF-*α*- and IL-1*β*-triggered activation of the NF-*κ*B signaling pathway

**Fig 1:**
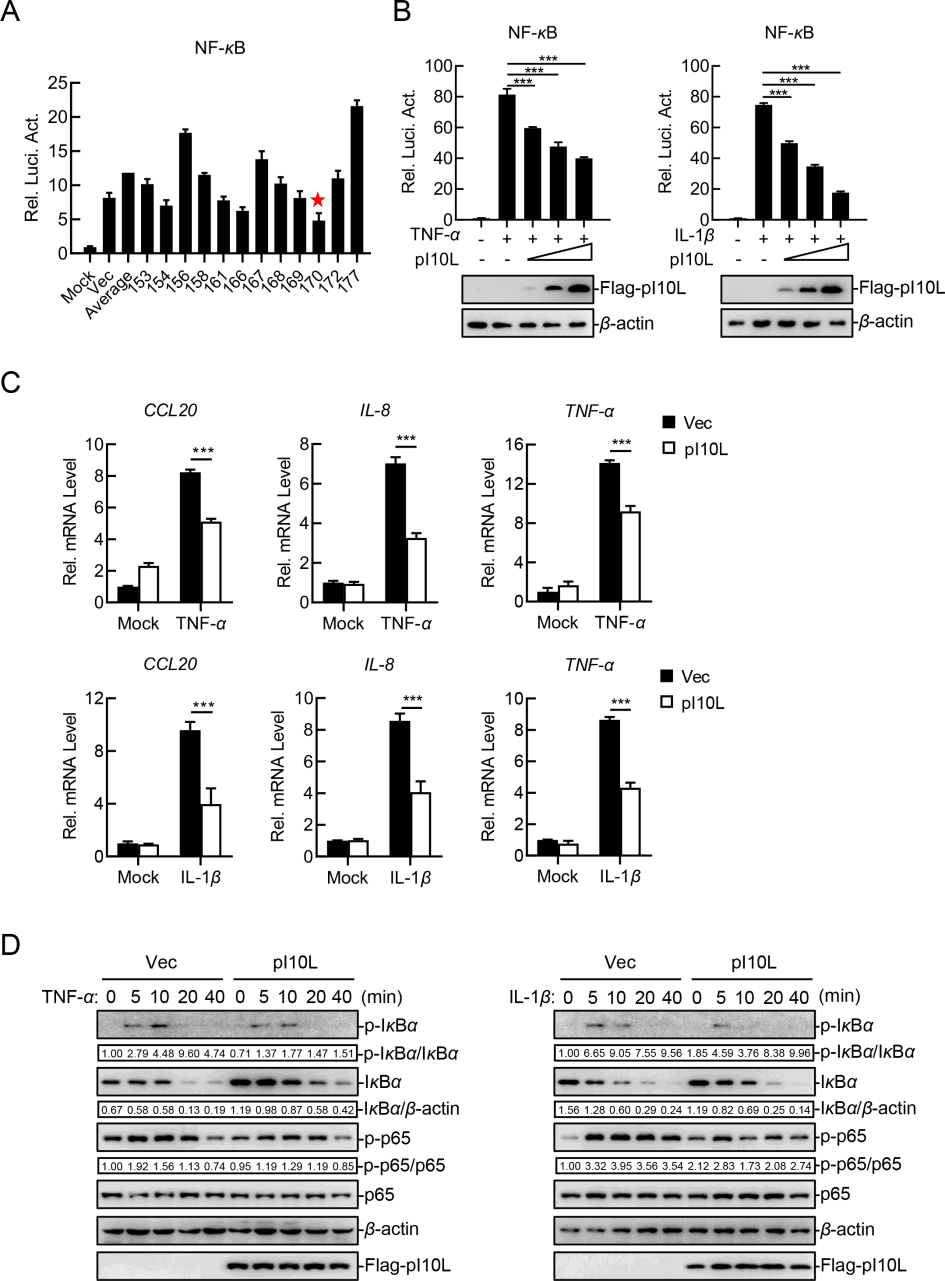
The ASFV I10L protein inhibits the TNF-*α*- and IL-1*β*-triggered NF-*κ*B signaling pathway in HEK293T cells. (**A**) The ASFV pI10L inhibits TNF-*α*-triggered activation of the NF-*κ*B promoter. HEK293T cells were transfected with pNF-*κ*B-Fluc (0.1 *µ*g), pRL-TK (0.01 *µ*g), and the plasmids expressing different ASFV proteins (0.2 *µ*g). Twenty hours later, the cells were treated with TNF-*α* (10 ng/mL) for 10 hours, after which the luciferase assays were performed. ★: pI10L. (**B**) The ASFV pI10L inhibits the TNF-*α*- and IL-1*β*-triggered activation of the NF-*κ*B promoter in a dose-dependent manner. HEK293T cells were transfected with the pI10L-expressing plasmid at different concentrations (0, 0.01, 0.02, or 0.04 *µ*g) along with pNF-*κ*B-Fluc (0.1 *µ*g) and pRL-TK (0.01 *µ*g). Twenty hours later, the luciferase assay and immunoblotting analysis were performed after the cells were treated with TNF-*α* (10 ng/mL) or IL-1*β* (10 ng/mL) for 10 hours. (**C**) The ASFV pI10L inhibits the TNF-*α*- and IL-1*β*-triggered the transcription of proinflammatory cytokines. HEK293T cells were transfected with either the pRK (Vec) or the pFlag-pI10L (0.2 *µ*g) for 20 hours, and RT-qPCR was performed following treatment with TNF-*α* (10 ng/mL) or IL-1*β* (10 ng/mL) for 6 hours. (**D**) The ASFV pI10L inhibits the TNF-*α*- and IL-1*β*-triggered phosphorylation of I*κ*B*α* and p65. HEK293T cells were transfected with either the Vec or the pFlag-pI10L (0.2 *µ*g) for 20 hours. The cells were then treated with TNF-*α* (20 ng/mL) or IL-1*β* (20 ng/mL) for 5, 10, or 20 minutes, followed by immunoblotting analysis. Densitometric analysis of protein expression level was performed with ImageJ software. The data shown are the mean ± SD from one representative experiment performed in triplicate (A to C). ***P < 0.001 (unpaired *t* test).

To identify candidate molecules involved in virus-induced inflammatory responses, we screened 179 ASFV proteins for their ability to regulate the TNF-*α*-triggered activation of the NF-*κ*B signaling in reporter assays. These efforts led to the identification of the ASFV pI10L (Fig. 1A). Further experiments in HEK293T cells indicated that pI10L inhibited the TNF-*α*- and IL-1*β*-triggered activation of the NF-*κ*B promoter in a dose-dependent manner (Fig. 1B). To investigate whether pI10L is involved in the regulation of endogenous NF-*κ*B signaling, we measured the transcription levels of several proinflammatory cytokines following treatment with TNF-*α* and IL-1*β* in the pI10L-expressing HEK293T cells. The ectopically expressed pI10L remarkably inhibited the mRNA transcription levels of the *CCL20, IL-8,* and *TNF-α* genes induced by TNF-*α* and IL-1*β*, as demonstrated by quantitative RT-PCR (RT-qPCR) (Fig. 1C). Consistently, the phosphorylation of I*κ*B*α* and p65 induced by TNF-*α* and IL-1*β*, which are the hallmarks of the activation of downstream signaling components, was remarkably inhibited in the pI10L-expressing HEK293T cells compared with the empty vector (Vec)-transfected cells (Fig. 1D). These results suggest that pI10L is involved in regulating the expression of proinflammatory cytokines triggered by TNF-*α* and IL-1*β*.

**Fig 2:**
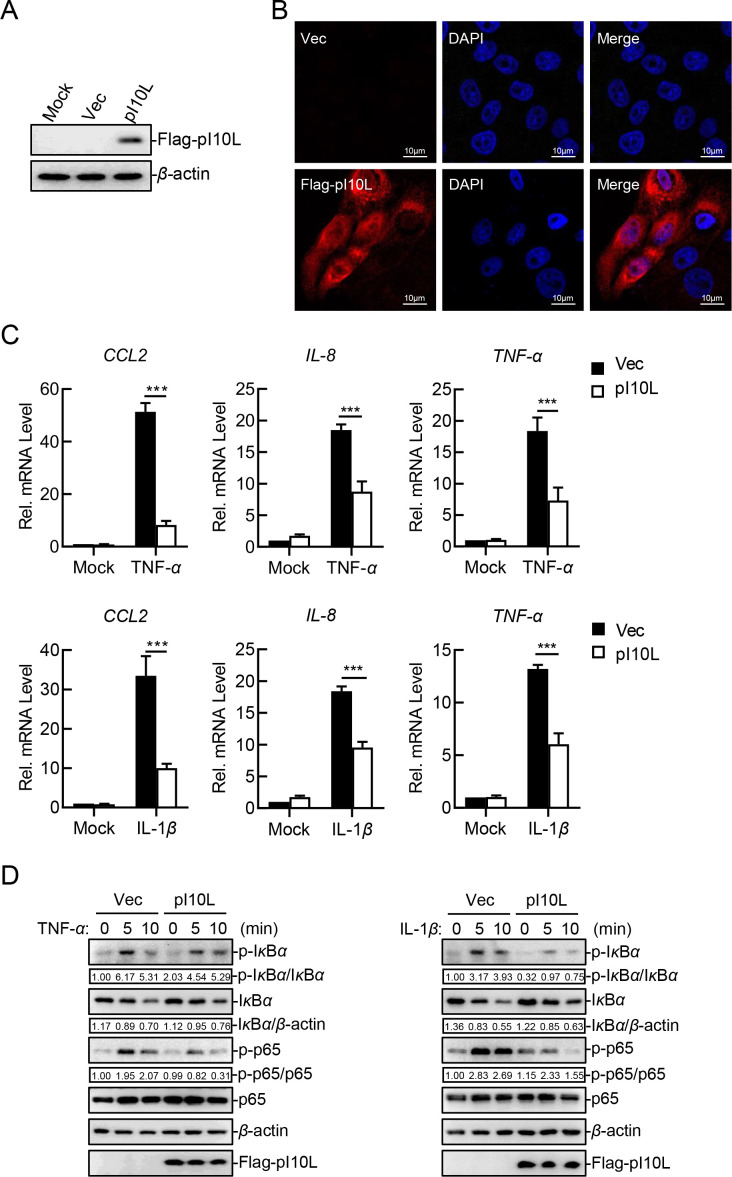
The ectopically expressed I10L protein inhibits the TNF-*α*- and IL-1*β*-triggered NF-*κ*B signaling pathway. (**A**) PK-15 cell lines stably expressing the ASFV pI10L were successfully established. The PK-15 cell lines stably expressing the ASFV I10L protein or harboring the empty vector were lysed and the expression of pI10L was measured through immunoblotting. (**B**) The ASFV pI10L is localized in both the cytoplasm and the nucleus of the infected cells. The PK-15 cell lines stably expressing the ASFV pI10L or harboring Vec were fixed with 4% paraformaldehyde and subjected for confocal microscopy. (**C**) The ASFV pI10L inhibits the TNF-*α*- and IL-1*β*-triggered transcription of proinflammatory cytokines in PK-15 cells. The PK-15 cell lines stably expressing the ASFV pI10L or harboring Vec were treated with TNF-*α* (10 ng/mL) or IL-1*β* (10 ng/mL) for 10 hours, and then total RNA was prepared for RT-qPCR assay. (**D**) The ASFV pI10L inhibits the TNF-*α*- and IL-1*β*-triggered phosphorylation of I*κ*B*α* and p65 in PK-15 cells. The PK-15 cell lines stably expressing the ASFV pI10L or harboring Vec were treated with TNF-*α* (20 ng/mL) or IL-1*β* (20 ng/mL) for 5 or 10 minutes, after which the cells were lysed and immunoblotting analysis was performed. Densitometric analysis of protein expression level was performed with ImageJ software. Data shown are the mean ± SD from one representative experiment performed in triplicates. ***P < 0.001 (unpaired *t* test).

Considering that pigs are the only mammalian species known to be susceptible to ASFV, a PK-15 cell line stably expressing pI10L was established to further identify its function. Immunoblotting analysis demonstrated that pI10L was expressed in PK-15 cells (Fig. 2A). Confocal microscopy indicated that pI10L was expressed in both the cytoplasm and nucleus (Fig. 2B). In agreement with results from HEK293T cells, RT-qPCR results showed that the ectopically expressed pI10L dramatically inhibited the TNF-*α*- and IL-1*β*-induced transcription of the *CCL2, IL-8*, and *TNF-α* genes in PK-15 cells (Fig. 2C). Moreover, pI10L reduced the phosphorylation of I*κ*B*α* and p65, followed by delayed degradation of I*κ*B*α* in PK-15 cells (Fig. 2D). Taken together, the results suggest that pI10L inhibits the TNF-*α*- and IL-1*β*-triggered NF-*κ*B signaling pathway.

### Deletion of the *I10L* gene from ASFV results in enhanced activation of the NF-*κ*B signaling pathway

**Fig 3:**
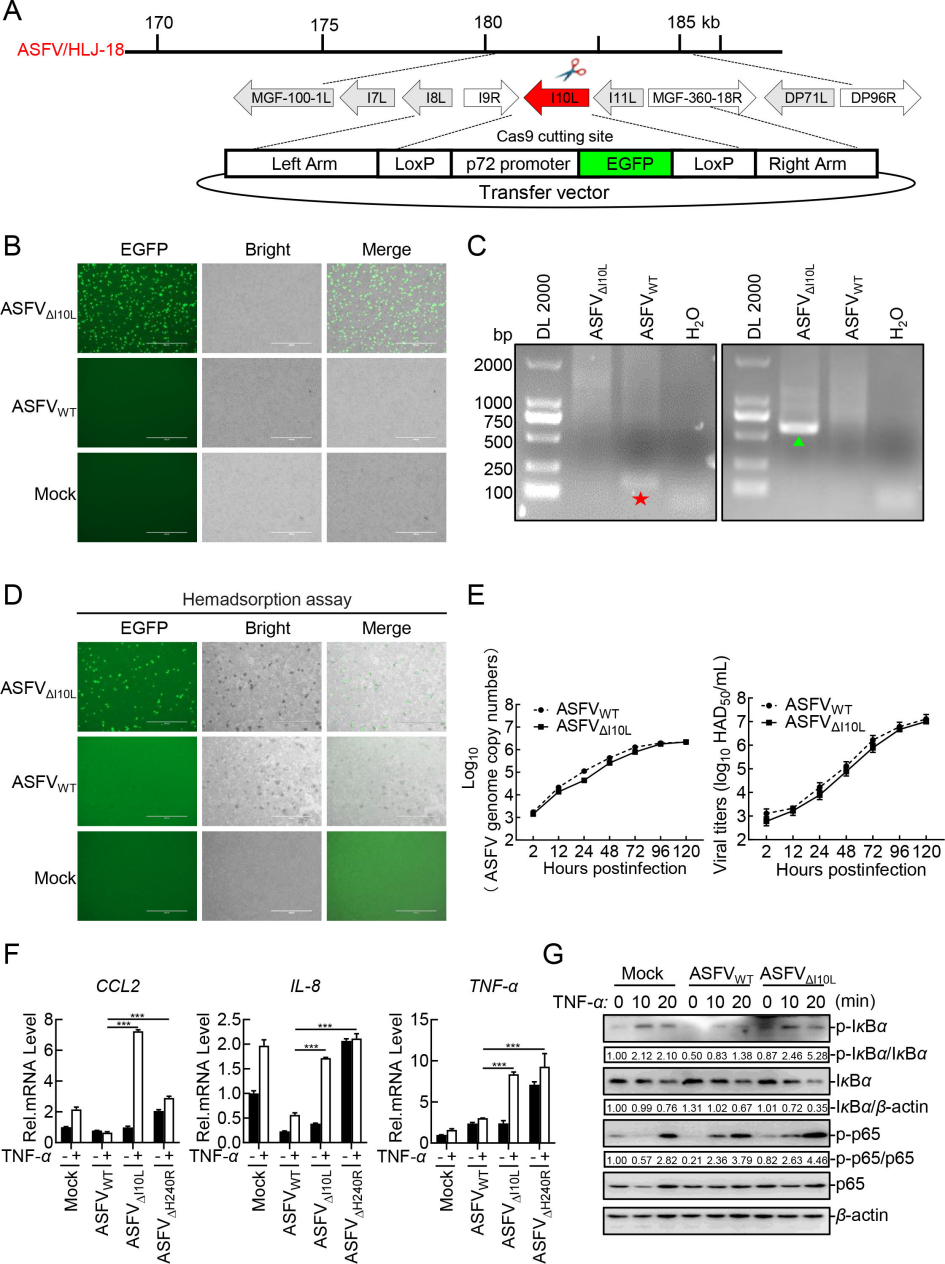
Deletion of the *I10L* gene from the ASFV genome results in enhanced activation of the NF-*κ*B signaling pathway. (**A**) Schematic diagram of the genome structure of ASFV_ΔI10L_. (**B**) Purification of ASFV_ΔI10L_ in primary porcine alveolar macrophages (PAMs). The recombinant ASFV_ΔI10L_ was screened out relying on EGFP fluorescence detection. (**C**) Identification of ASFV_ΔI10L_ by PCR. Specific primers of *I10L* (★) and *EGFP* (▲) were used for PCR, and the samples were subjected to agarose gel electrophoresis. (**D**) Hemadsorption characteristics of ASFV_ΔI10L_. PAMs were infected with ASFV_ΔI10L_ or ASFV_WT_ or left uninfected serving as a negative control. Hemadsorption and fluorescence were examined by microscopy. (**E**) *In vitro* replication characteristics of ASFV_ΔI10L_. Left panel: genome copies of ASFV_ΔI10L_ or ASFV_WT_; Right panel: viral titers of ASFV_ΔI10L_ or ASFV_WT_. (**F**) ASFV_ΔI10L_ induces much higher levels of *CCL2*, *IL-8*, and *TNF-α* than did ASFV_WT_. PAMs were either mock-infected or infected with ASFV_WT_, ASFV_ΔI10L_ or ASFV_ΔH240R_ at a multiplication of infection (MOI) of one. Forty-eight hours later, the cells were treated with TNF-*α* (10 ng/mL) (or left untreated) for another 20 minutes. Total RNA was extracted and subjected to RT-qPCR. (**G**) ASFV_ΔI10L_ elevates the phosphorylation levels of I*κ*B*α* and p65. PAMs were left uninfected or infected with ASFV_WT_ or ASFV_ΔI10L_ (MOI = 3) for 24 hours. The cells were then treated or untreated with TNF-*α* (20 ng/mL) for 10 or 20 minutes, immunoblotting analysis was performed. Densitometric analysis of protein expression levels were performed using ImageJ software. Data shown are the mean ± SD from one representative experiment performed in triplicates. ***P < 0.001 (unpaired *t* test).

To characterize the functional role of pI10L in the regulation of the NF-*κ*B signaling pathway, recombinant ASFV_ΔI10L_ was generated from highly virulent ASFV_WT_ by homologous recombination. The *I10L* gene was replaced with a fluorescent gene EGFP-containing cassette under the control of the ASFV p72 promoter (Fig. 3A). ASFV_ΔI10L_ was generated and purified after 10 rounds of fluorescence screening (Fig. 3B). The recombinant ASFV without parental ASFV contamination was confirmed by diagnostic PCR (Fig. 3C). The results of hemadsorption assay revealed “rosettes” of red blood cells on the ASFV_ΔI10L_- or ASFV_WT_-infected PAMs, indicating that the deletion of *I10L* did not affect the hemadsorption property of ASFV (Fig. 3D). Furthermore, the growth kinetics of ASFV_ΔI10L_ were similar to those of ASFV_WT_ depending on the time point considered (Fig. 3E), indicating that the *I10L* gene deletion did not affect the replication of ASFV in PAMs.

To verify the function of pI10L, we next examined the abilities of ASFV_ΔI10L_ in regulating the activation of the NF-*κ*B signaling pathway. ASFV_ΔH240R_, a recombinant ASFV lacking the *H240R* gene (23) was included as a positive control to validate the results. Following infection of PAMs with ASFV_ΔI10L_, ASFV_ΔH240R_, or ASFV_WT_ at a multiplication of infection (MOI) of one for 48 hours, the PAMs were treated with TNF-*α* or RPMI-1640 medium for another 20 minutes. As shown in the Fig. 3F, the deletion of the *I10L* or *H240R* gene resulted in enhanced transcription of proinflammatory cytokines compared with ASFV_WT_ upon treatment with TNF-*α* in PAMs, and the data of pH240R were consistent with the previous studies (23, 24, 30). Consistently, the ASFV_ΔI10L_-infected PAMs showed elevated phosphorylation levels of I*κ*B*α* and p65 (Fig. 3G). These results suggest that pI10L plays an important role in the evasion of antiviral responses to ASFV.

### The ASFV pI10L is associated with IKK*β*, NEMO, and NF-*κ*B

**Fig 4:**
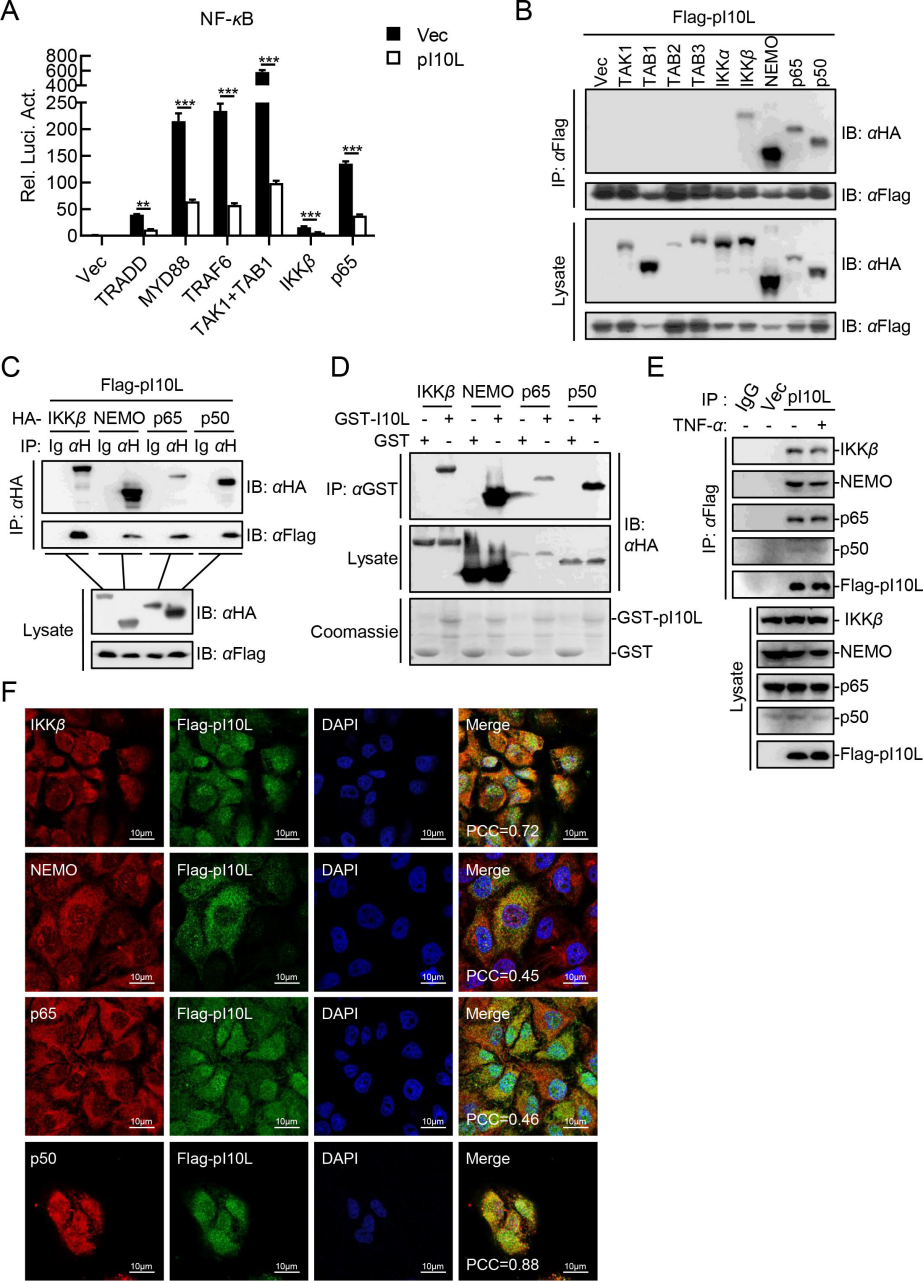
The ASFV I10L protein interacts with IKK*β*, NEMO and NF-*κ*B. (**A**) The ASFV pI10L functions at or upstream of p65. HEK293T cells were co-transfected with pNF-*κ*B-Fluc (0.1 *µ*g), pRL-TK (0.01 *µ*g), the plasmids expressing the indicated proteins (TRADD, MYD88, TRAF6, TAK1, TAB1, IKK*β*, and p65) (0.05 *µ*g), the empty vector or pFlag-pI10L (0.04 *µ*g) for 20 hours, followed by luciferase assay. (**B and C**) The ASFV pI10L interacts with IKK*β*, NEMO, p65, and p50. HEK293T cells were transfected with the plasmids pHA-IKK*β*, -NEMO, -p65, or -p50, and pFlag-pI10L for 20 hours, and then lysed for co-IP with anti-HA or anti-Flag MAb, followed by immunoblotting analysis. (**D**) The ASFV pI10L binds to IKK*β*, NEMO, p65, and p50 *in vitro*. The recombinant GST-tagged pI10L was incubated with the HA-tagged IKK*β*, NEMO, p65, or p50, followed by immunoblotting analysis. (**E**) The ASFV pI10L interacts with the endogenous IKK*β*, NEMO, p65, and p50. The PK-15 cells stably expressing pI10L were left untreated or treated with TNF-*α* (20 ng/mL) for 30 minutes and then lysed for co-IP with anti-Flag MAb, followed by immunoblotting analysis. (**F**) The ASFV pI10L were co-localized with IKK*β*, NEMO, p65, and p50 mainly in the cytoplasm. The PK-15 cells stably expressing the ASFV pI10L were fixed with 4% paraformaldehyde and subjected to confocal microscopy. The Pearson’s correlation coefficient (PCC) was used to indicate the co-localization between IKK*β*, NEMO, p65, or p50 (red) and Flag-pI10L (green). Data shown are the mean ± SD from one representative experiment performed in triplicates. ***P < 0.001 (unpaired *t* test).

It has been previously reported that pI10L functions in the TNF-*α*- and IL-1*β*-triggered NF-*κ*B signaling, suggesting that this protein probably works at TAK1/TABs complex or its downstream level, which is the convergence of two pathways (19). To identify the targets involved in pI10L function, various components involved in NF-*κ*B signaling (including TRADD, MYD88, TRAF6, TAK1, TAB1, IKK*β*, and p65) were co-expressed with pI10L. As shown in Fig. 4A, pI10L inhibited the activation of the NF-*κ*B promoter mediated by all tested molecules upstream of p65, indicating that pI10L may function at the p65 level. In addition, transient transfection and co-immunoprecipitation (co-IP) experiments confirmed that pI10L interacted with IKK*β*, NEMO, p65, and p50, but not with TAK1, TAB2, TAB3, or IKK*α* (Fig. 4B and C). The results of GST pulldown assays further confirmed that pI10L was directly associated with IKK*β*, NEMO, p65, and p50 directly *in vitro* (Fig. 4D). In addition, endogenous co-IP experiments indicated that pI10L was constitutively associated with IKK*β*, NEMO, p65, and p50 in PK-15 cells. Moreover, this interaction was not affected by TNF-*α* (Fig. 4E). Consistently, confocal microscopy showed that pI10L co-localized with IKK*β*, NEMO, p65, and p50 mainly in the cytoplasm (Fig. 4F). These results suggest that pI10L is associated with IKKs and NF-*κ*B, and may function at the p65 level.

### The ASFV I10L protein regulates the activation and the association of IKK*β* with I*κ*B*α* and p65

**Fig 5:**
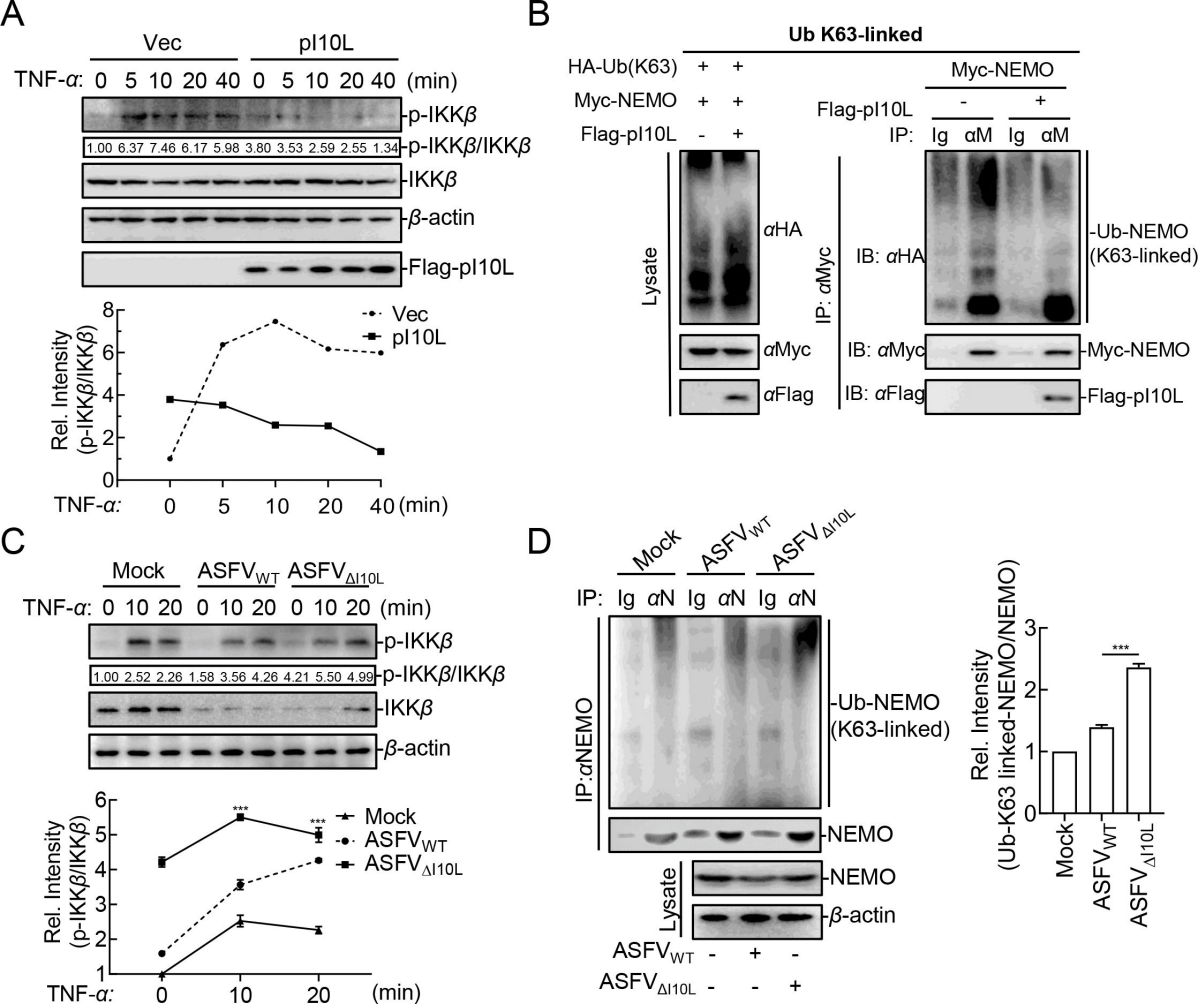
The ASFV I10L protein inhibits the phosphorylation of IKK*β* by reducing the K63-linked ubiquitination of NEMO. (**A**) The ASFV pI10L inhibits the TNF-*α*-triggered phosphorylation of IKK*β*. HEK293T cells were transfected with either the pRK (Vec) or the pFlag-pI10L plasmid. The cells were then treated with TNF-*α* (20 ng/mL) for 5, 10, 20, or 40 minutes, followed by immunoblotting analysis. Densitometric analysis of protein expression level was performed with ImageJ software. (**B**) The ASFV pI10L inhibits the K63-linked ubiquitination of NEMO. HEK293T cells were transfected with the plasmids expressing HA-K63O, Myc-NEMO, or Flag-pI10L, followed by co-IP and immunoblotting analysis. (**C**) ASFV_ΔI10L_ promotes the phosphorylation of IKK*β*. The primary porcine alveolar macrophages (PAMs) were left uninfected or infected with ASFV_WT_ or ASFV_ΔI10L_ (MOI = 3) for 24 hours, after which the cells were treated or untreated with TNF-*α* (20 ng/mL) for 10 or 20 minutes and immunoblotting analysis was performed. Densitometric analysis of protein expression levels were performed with ImageJ software. (**D**) ASFV_ΔI10L_ enhances the K63-linked ubiquitination of NEMO. PAMs were left uninfected or infected with ASFV_WT_ or ASFV_ΔI10L_ (MOI = 3) for 24 hours, and the cells were then treated or untreated with TNF-*α* (20 ng/mL) for 20 minutes, after which co-IP and immunoblotting analysis were performed. Densitometric analysis of protein expression levels were performed with ImageJ software. Data shown are the mean ± SD from one representative experiment performed in triplicates. ***P < 0.001 (unpaired *t* test).

IKK*β* is the principal kinase responsible for phosphorylating I*κ*B*α* (31) and p65 (32), and IKK*β* phosphorylation plays a central role in IKK complex activation. This study indicated that the TNF-*α*-triggered phosphorylation of IKK*β* was significantly prevented by the ectopically expressed pI10L (Fig. 5A). It has been reported that NEMO, the regulatory subunit of IKK complex, can be ubiquitinated by TRAF6 and TRAF2/5, and this K63-linked polyubiquitination is essential for the activation of IKK*β* (33). To determine how pI10L regulates IKK*β* activation, the K63-linked ubiquitination (Ub) of NEMO was examined. Western blot analysis showed that Ub-K63 linked conjugation to NEMO was decreased in the presence of pI10L (Fig. 5B). Compared with the parental virus ASFV_WT_, ASFV_ΔI10L_ infection consistently and remarkably enhanced the phosphorylation levels of IKK*β* (Fig. 5C) and the K63-linked ubiquitination of NEMO (Fig. 5D) upon TNF-*α* treatment. This suggests that the pI10L inhibits the IKK*β* phosphorylation by reducing the K63-linked ubiquitination of NEMO.

**Fig 6:**
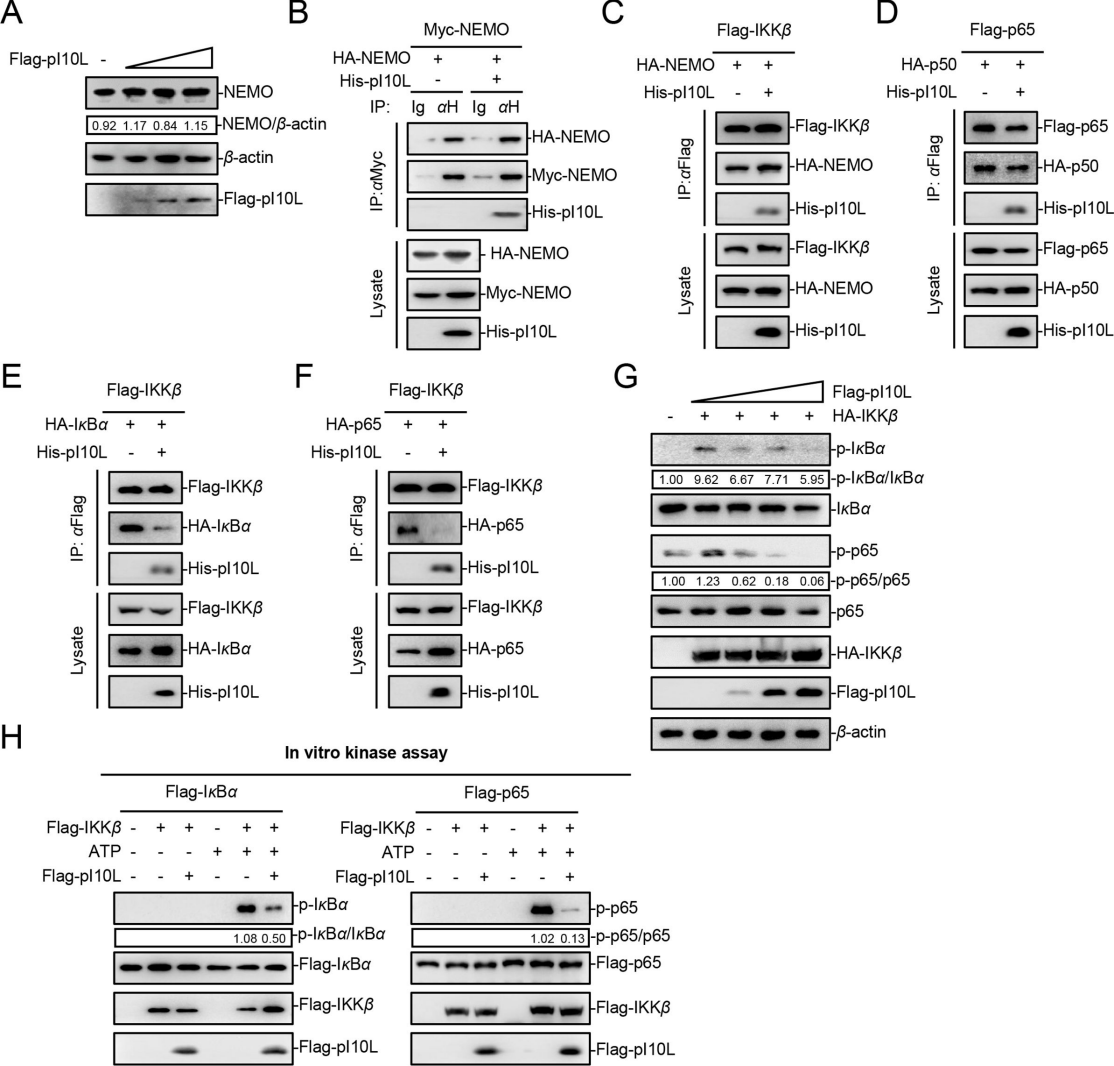
The ASFV I10L protein (pI10L) regulates the formation of the IKK*β*-I*κ*B*α*-p65 complex. (**A**) The ASFV pI10L has no effect on the expression of NEMO. HEK293T cells were transfected with various concentrations of the pI10L-expressing plasmid followed by immunoblotting analysis. (B–F) The ASFV pI10L inhibits the interaction between IKK*β* and I*κ*B*α* or p65, but does not regulate the assembly of the NF-*κ*B or IKK complex. HEK293T cells were transfected with the plasmids: pHA-NEMO, -p65, or -p50, pHis-pI10L, pMyc-NEMO, pFlag-IKK*β*, or -p65 for 24 hours. Myc-NEMO, Flag-IKK*β* or Flag-p65 was then used as bait protein to perform co-IP and immunoblotting analysis. (**G**) The ASFV pI10L inhibits the IKK*β*-mediated phosphorylation of I*κ*B*α* and p65 in a dose-dependent manner. HEK293T cells were transfected with various concentrations of the pI10L-expressing plasmid (0, 0.1, 0.2, or 0.4 *µ*g) and the IKK*β*-expressing plasmid (0.1 *µ*g). Twenty hours later, the cells were collected, and immunoblotting analysis was performed. (**H**) The ASFV pI10L suppresses the IKK*β* kinase activity towards I*κ*B*α* and p65. The Flag-tagged IKK*β*, I*κ*B*α*, p65, or pI10L were purified and subjected to *in vitro* kinase assay, followed by immunoblotting analysis. Densitometric analysis of the protein expression level was performed with ImageJ software.

It has been shown that the assembly of the IKK complex is indispensable for IKK*β* activation (34). The results demonstrated that pI10L had no effect on the expression or dimerization of NEMO (Fig. 6A and B), nor did it affect the association between IKK*β* and NEMO (Fig. 6C). Thus, pI10L does not regulate the assembly of the IKK complex assembly. Competitive binding experiments were subsequently carried out to explore whether pI10L affects the assembly of NF-*κ*B complexes, as well as the association between IKK*β* and its substrates I*κ*B*α* and p65. As shown in Fig. 6D, pI10L is not involved in the binding of p65 and p50. Interestingly, pI10L remarkably impaired the interaction between IKK*β* and I*κ*B*α* or p65 (Fig. 6E and F). In addition, upon treatment with increasing concentrations of pI10L, the IKK*β*-mediated phosphorylation of I*κ*B*α* and p65 was decreased in a dose-dependent manner (Fig. 6G). To investigate whether pI10L directly regulates the kinase activity of IKK*β*, an *in vitro* kinase assay was performed. Immunoblotting analysis showed that I*κ*B*α* and p65 were phosphorylated by IKK*β* in the presence of ATP, whereas the addition of pI10L dramatically impaired this phosphorylation process (Fig. 6H). Taken together, our findings show that pI10L functions by regulating K63-linked polyubiquitination of NEMO to suppress the activation of IKK*β*. Furthermore, pI10L is associated with IKK*β*, which in turn suppresses its kinase activity towards I*κ*B*α* and p65, thereby inhibiting the activation of the NF-*κ*B signaling pathway.

### The ASFV I10L protein inhibits the nuclear translocation of p65

**Fig 7:**
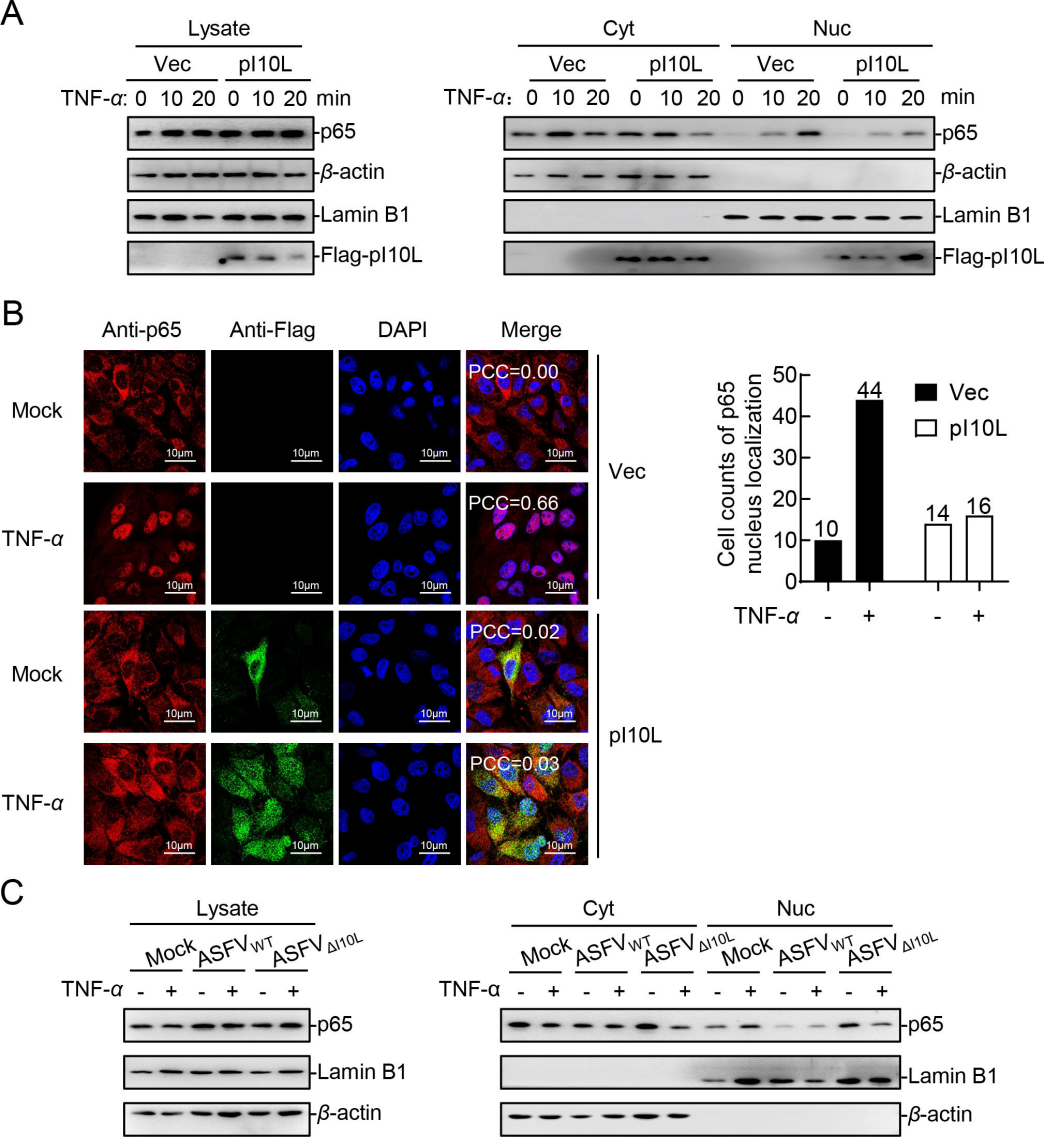
The ASFV I10L protein inhibits the nuclear translocation of p65. (**A**) The ASFV pI10L reduces the distribution of p65 in the nucleus. The PK-15 cell lines stably expressing the ASFV pI10L or harboring the empty vector were treated with TNF-*α* (20 ng/mL) for 10 or 20 minutes. The cells were next concentrated and lysed, followed by subcellular fractionation assay and immunoblotting analysis. (**B**) The ASFV pI10L inhibits the TNF-*α*-triggered nuclear translocation of p65. The PK-15 cell lines stably expressing the ASFV pI10L or harboring the empty vector were treated with TNF-*α* (10 ng/mL) or PBS for 5 minutes and then subjected to confocal microscopy. The PCC was used to indicate the overlapping coefficient between p65 (red) and nuclei (blue). A total of 50 cells were randomly selected to evaluate the nuclear localization ratio of p65, and the statistical analysis was carried out using GraphPad Prism. (**C**) Deletion of the *I10L* gene greatly compromises the ability of ASFV to block the activation of the NF-*κ*B signaling pathway. The primary porcine alveolar macrophages were left uninfected or infected with ASFV_WT_ or ASFV_ΔI10L_ (MOI = 3) for 24 hours, after which the cells were treated or untreated with TNF-*α* (20 ng/mL) for 20 minutes, subcellular fractionation assay, and immunoblotting analysis were performed.

It has been shown that the nuclear translocation of p65, which is released after I*κ*B*α* is degraded upon phosphorylation by IKK*β*, is an indicator of the NF-*κ*B signaling activation (20). Therefore, we examined whether p65 is translocated to the nucleus in the presence of pI10L. The results of subcellular fractionation assay and confocal microscopy analysis showed that the TNF-*α*-induced nuclear translocation of p65 was inhibited in the pI10L-expressing cells (Fig. 7A and B). Furthermore, the results indicated that the deficiency of pI10L attenuated the ability of ASFV to antagonize the TNF-*α*-induced nuclear translocation of p65 (Fig. 7C). The results indicated that the deletion of pI10L weakened the ability of ASFV to block the activation of the NF-*κ*B signaling pathway.

### Amino acids 1–102 on pI10L are essential for suppressing NF-*κ*B activation

**Fig 8:**
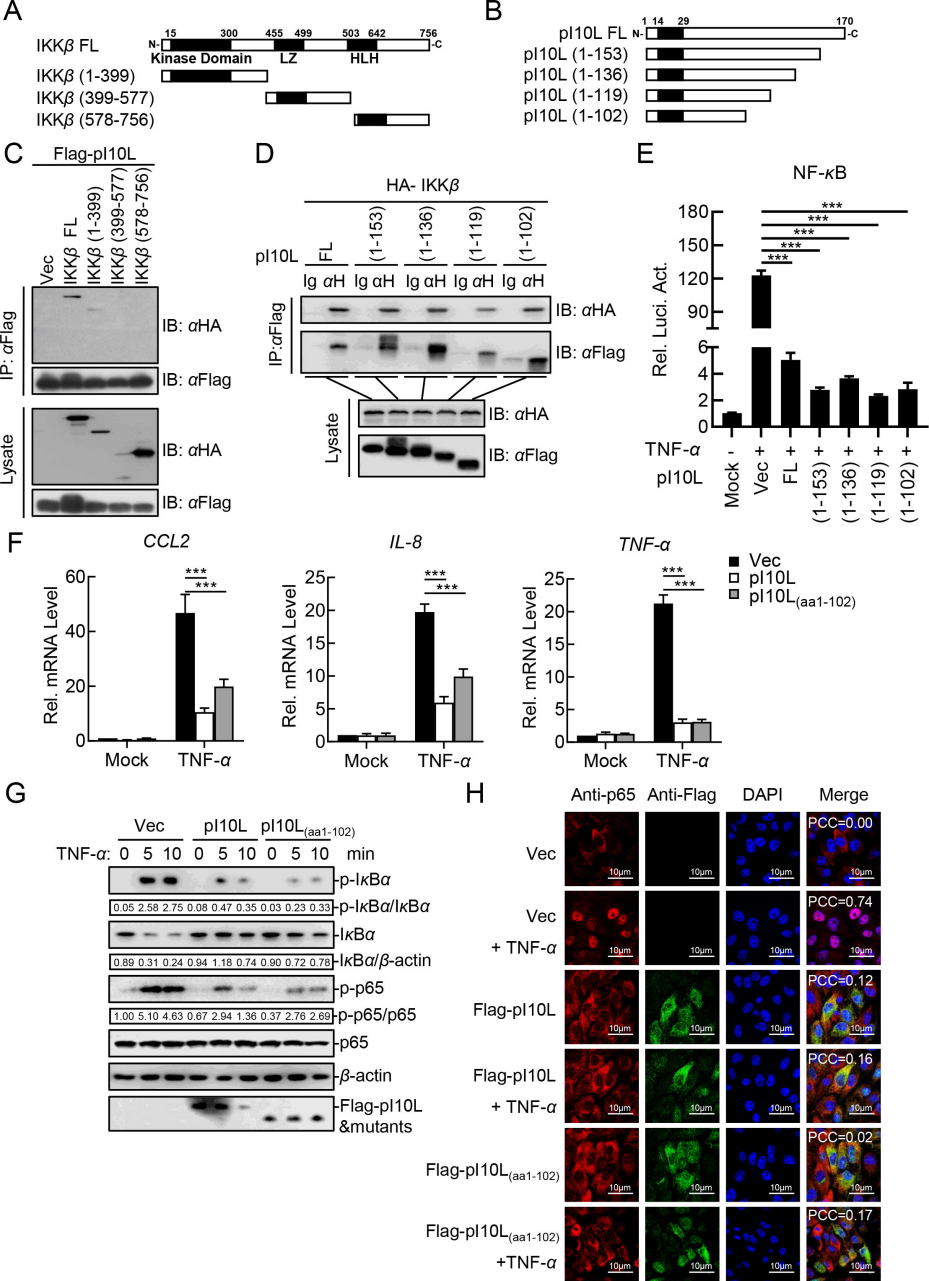
The aa 1–102 of the ASFV I10L protein are essential for suppressing the activation of the NF-*κ*B signaling pathway. (**A**) Schematic illustration of the truncated IKK*β* mutants. (**B**) Schematic illustration of the truncated pI10L mutants. (**C**) The ASFV pI10L interacts with the kinase domain of IKK*β*. The plasmids expressing the complete or truncated IKK*β* were co-transfected with the pI10L-expressing plasmid for 24 hours, after which Flag-pI10L was used as a bait to perform co-IP and immunoblotting analysis. (**D**) The truncated pI10L containing aa 1–102 is essential for interacting with IKK*β*. The plasmids expressing the complete or truncated pI10L were co-transfected with IKK*β* for 24 hours, after which the complete and truncated Flag-pI10L were used as baits to perform co-IP and immunoblotting analysis. (**E**) The ASFV pI10L(aa1-102) is essential for inhibiting the TNF-*α*-triggered activation of the NF-*κ*B promoter. HEK293T cells were transfected with pNF-*κ*B-Fluc (0.1 *µ*g), pRL-TK (0.01 *µ*g), and the plasmids expressing the complete or truncated pI10L (0.04 *µ*g) for 20 hours followed by luciferase assay. (**F**) The ASFV pI10L(aa1-102) dramatically reduces the TNF-*α*-triggered transcription of the *CCL2, IL-8*, and *TNF-α* genes. The PK-15 cell lines stably expressing the ASFV pI10L or pI10L(aa1-102) or harboring the empty vector were treated with TNF-*α* (10 ng/mL) or IL-1*β* (10 ng/mL) for 10 hours, and then total RNA was prepared for RT-qPCR analysis. (**G**) The ASFV pI10L(aa1-102) dramatically reduces the TNF-*α*-triggered phosphorylation of I*κ*B*α* and p65. The PK-15 cells stably expressing the ASFV pI10L or pI10L(aa1-102) or harboring the empty vector were treated with TNF-*α* (20 ng/mL) for 5 or 10 minutes and then lysed for immunoblotting analysis. (**H**) The ASFV pI10L(aa1-102) inhibits the TNF-*α*-triggered nuclear translocation of p65. HEK293T cells were transfected with Vec or the pI10L- or pI10L(aa1-102)-expressing plasmid. After 20 hours, the cells were treated with TNF-*α* (10 ng/mL) for 10 minutes and then fixed for immunostaining followed by confocal microscopy. Data shown are the mean ± SD from one representative experiment performed in triplicates. (**E and F**) ***P < 0.001 (unpaired *t* test). FL, full-length; LZ, leucine zipper domain; HLH, helix-ring-helix domain.

To investigate the crucial regions in IKK*β* and pI10L responsible for their interaction, as well as the suppression of NF-*κ*B activation, we constructed a series of plasmids expressing the complete or truncated IKK*β* (Fig. 8A) and pI10L (Fig. 8B). Domain mapping analysis revealed that the kinase domain of IKK*β* was responsible for its interaction with pI10L (Fig. 8C). Consistently, the truncated pI10L containing aa 1–102 was sufficient to bind to IKK*β* (Fig. 8D). These results further confirm that the binding of pI10L to IKK*β* ultimately suppresses the catalytic activity of IKK*β*.

We next evaluated whether the aa 1–102 of pI10L are sufficient to inhibit the TNF-*α*-triggered activation of the NF-*κ*B signaling. In reporter assay, all the mutants containing aa 1–102 were found to remarkably inhibit the activation of the NF-*κ*B promoter (Fig. 8E). RT-qPCR experiments demonstrated that the ectopic expression of pI10L or pI10L(aa1-102) dramatically reduced the transcript level of the *CCL2*, *IL-8*, and *TNF-α* genes in PK-15 cells (Fig. 8F). Consistently, the phosphorylation of I*κ*B*α* and p65 (Fig. 8G), as well as the nuclear translocation of p65 (Fig. 8H), were remarkably impaired in the pI10L- or pI10L(aa1-102)-expressing PK-15 cells. Taken together, our results suggest that the domain defined by aa 1–102 on pI10L is essential for the suppression of NF-*κ*B activation.

## DISCUSSION

The innate immune response is the first line of host defense against viral infection and is initiated upon sensing conserved viral structural components called pathogen-associated molecular patterns (PAMPs) by pattern recognition receptors (PRRs) in host cells (35 – 37). The sensing of viral PAMPs by PRRs activates a series of signaling events, leading to the expression of downstream antiviral effector proteins including type I interferons and proinflammatory cytokines (37 – 39). ASFV is a giant, complex DNA virus that encodes more than 160 proteins required for successful infection, thereby enabling replication and immune evasion *in vivo*. Previous studies have demonstrated that ASFV infection activates antiviral signaling pathways with increased expression levels of interferon-stimulated genes and proinflammatory cytokines (15), especially the NF-*κ*B signaling (40). However, the mechanisms of ASFV immune evasion remain unclear. In this study, we identified the ASFV pI10L as an inhibitor of virus-induced inflammatory responses following viral infection and treatment with TNF-*α* or IL-1*β*.

We found that transient transfection of *I10L* in HEK293T cells remarkably inhibited the activation of the NF-*κ*B promoter, transcription of proinflammatory cytokines, and the phosphorylation of I*κ*B*α* and p65 induced by TNF-*α* or IL-1*β*. We obtained the same results as in the PK-15 cells stably expressing pI10L. These findings suggest that pI10L plays an important role in the immune escape of ASFV in different cell types.

Considering the unbiased effects on the TNF-*α*- and IL-1*β*-triggered NF-*κ*B signaling, we hypothesized that pI10L functions at the level of or downstream of the TAK1-TABs complex. Interestingly, pI10L inhibited p65 and all the tested proteins upstream of the p65-mediated activation of the NF-*κ*B signaling pathway. In addition, pI10L was found to be associated with IKK*β*, NEMO, and NF-*κ*B. There are several possible mechanisms underlying how pI10L functions. Firstly, pI10L inhibits the activation of IKK*β* by impairing the assembly of the IKK complex or the polyubiquitination of NEMO. Secondly, it is possible that pI10L suppresses the binding of p65 to p50. Thirdly, pI10L could block the activation and nuclear translocation of p65. The results demonstrated that pI10L inhibits the phosphorylation of IKK*β* by regulating the K63-linked ubiquitination of NEMO, as well as reduced the catalytic activity of towards I*κ*B*α* and p65 by interacting with IKK*β*. However, pI10L only slightly interfered with the assembly of the NF-*κ*B or IKK complex.

It has been reported that I*κ*B*α* and p65 are phosphorylated by IKK*β* in the TNF-*α*- and IL-1*β*-triggered NF-*κ*B signaling pathway (41). Therefore, we performed kinase assays *in vitro*. The results confirmed that IKK*β* could phosphorylate I*κ*B*α* and p65, but this effect was remarkably inhibited by pI10L, owing to the fact that pI10L interrupted the binding of I*κ*B*α* and p65 to IKK*β*. Using subcellular fractionation assay and confocal microscopy analysis, we found that ectopically expressed pI10L dramatically inhibited the translocation of p65 to the nuclear. Domain mapping analysis indicated that pI10L interacts with the kinase domain of IKK*β* through its N-terminal (aa 1–102). Together, these findings demonstrate that pI10L inhibits IKK*β* kinase activity through direct association with IKK*β*, obstructing its catalytic center, or by competing with I*κ*B*α* and p65 as a substrate. Confirming the exact mechanism will be a very interesting direction for future investigation.

Furthermore, the effects of pI10L on the expression of proinflammatory cytokines were explored using the *I10L* gene-deleted ASFV. The results showed that the hemadsorption and replication of ASFV were slightly affected by pI10L in PAMs. However, ASFV_ΔI10L_ induced higher activation of the NF-*κ*B signaling pathway than did ASFV_WT_. To date, several other ASFV-encoded proteins that regulate the activation of NF-*κ*B have been identified, such as the pL83L (26), pMGF505-7R (22, 42), and the H240R protein (pH240R) (23, 30). ASFV lacking these genes induces decreased expression of various proinflammatory cytokines and increased viral replication, whereas the relationship among these regulators remains unclear. Single gene deletion hardly affects the virulence of ASFV (except for pMGF505-7R and pH240R), which may be because these proteins function in concert during viral infection and pathogenesis. For example, the proteins may participate in the inflammatory response during different stages of viral infection. To hasten the development of an effective vaccine against ASF, further in-depth studies are needed to explore how these proteins work together.

**Fig 9:**
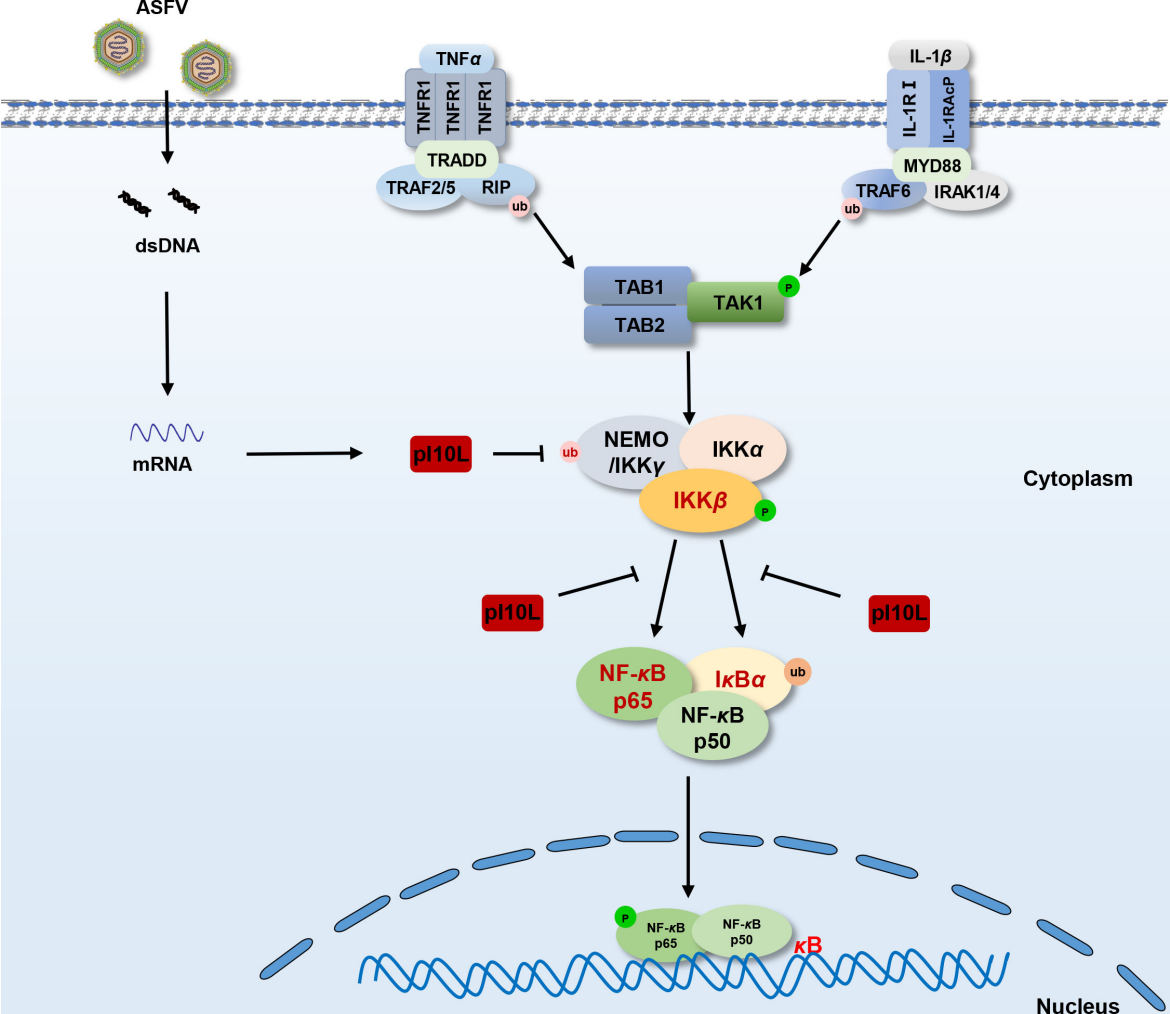
Schematic diagram for the mechanism by which the ASFV I10L protein suppresses the host cell proinflammatory responses. The ASFV pI10L can inhibit the TNF-*α*- and IL-1*β*-triggered inflammatory responses by targeting IKK*β*. pI10L inhibits the phosphorylation of IKK*β* by reducing the K63-linked ubiquitination of NEMO and hinders the association of IKK*β* with its substrates I*κ*B*α* and p65, leading to reduced phosphorylation of I*κ*B*α* and p65, as well as the nuclear translocation of p65, and subsequently the expression of proinflammatory cytokines.

In conclusion, as illustrated in Fig. 9, our findings demonstrated a critical role of pI10L in regulating the TNF-*α*- and IL-1*β*-triggered NF-*κ*B signaling by targeting IKK*β*. As the inflammatory response plays an important role in the pathogenesis of ASF (15, 16) and host antiviral innate immunity, the identification of pI10L helps clarify the complicated immune evasion mechanisms of ASFV and may lead to the discovery of potential targets for the development of novel ASF vaccines or antivirals.

## MATERIALS AND METHODS

### Reagents, cells, and viruses

Recombinant human TNF-*α* (catalog no. 300-01A) and IL-1*β* (catalog no. 200-01B) were purchased from Peprotech (Cranbury, NJ, USA). HEK293T cells were kindly provided by Dr. Hong-Bing Shu, and PK-15 cells were obtained from the ATCC. The ASFV HLJ/2018 strain was isolated from field samples in China as previously described (GenBank accession no. MK333180.1)

### Construction of plasmids

The plasmids expressing the HA-, Flag-, or His-tagged pI10L, TRADD, MYD88, TAK1, TAB1, TAB2, TAB3, IKK*α*, IKK*β*, NEMO, I*κ*B*α*, p65, p50, and their mutants were constructed by standard molecular biology techniques. The stable cell lines stabling expressing pI10L or its truncated mutants were established using the lentivirus-mediated gene-editing technology (43). Briefly, HEK293T cells were transfected with the packaging plasmids psPAX2 and pMD2.0G, recombinant plasmids, or the empty vector pLOV. After 36–48 hours, the pseudovirus-containing culture supernatant was harvested to infect PK-15 cells in the presence of polybrene (8 *µ*g/mL). The infected cells were screened with puromycin (3 *µ*g/mL) for 6 days to establish stable cell lines. The primers used in this study are listed in Table 1.

**TABLE 1:** Primers used for PCR in this study

Plasmids	Primers (5’−3’)
pFlag-pI10L	F: CCGGGATTTGGATCCATGTTTTATCCTGTTGTTCA R: CTTGTAGTCGCTAGCAAGTACATTTCTGGCTATTC
pFlag-pI10L(aa 1–102)	F: CCGGGATTTGGATCCATGTTTTATCCTGTTGTTCA R: CTTGTAGTCGCTAGCTATACGTGTTTTTCCAAATG

### Transfection and reporter assays

HEK293T and PK-15 cells were transfected using the standard polyetherimide method. In the reporter assays, an empty control plasmid was added separately to ensure the same amount of total DNA was present in a simple sample. Luciferase assays were performed using a dual-specific luciferase assay kit (catalog no. E1910; Promega). The data shown are the *firefly* luciferase activity levels of the indicated samples normalized to *Renilla* luciferase activity.

### RT-qPCR

Total RNA was extracted using the TRIzol Reagent (catalog no. 9108; TaKaRa) and reverse-transcribed to cDNA using the HiScript III 1st Strand cDNA Synthesis Kit (catalog no. R312-01; Vazyme) according to the manufacturer’s protocol. qPCR was performed in triplicate using HiScript II Q RT SuperMix (catalog no. R223-01; Vazyme) according to the manufacturer’s protocol. Data shown are the relative abundance of the indicated mRNAs normalized to those of GAPDH. The primers used for RT-qPCR are listed in Table 2.

**TABLE 2:** Primers used for RT-qPCR in this study

Gene name	Sequence (5’−3’)
h-*GAPDH*	F:GACAAGCTTCCCGTTCTCAG R:GAGTCAACGGATTTGGTGGT
h-*CCL20*	F:AAGTTGTCTGTGTGCGCAAATCC R:CCATTCCAGAAAAGCCACAGTTTT
h-*IL8*	F:GAGAGTGATTGAGAGTGGACCAC R:CACAACCCTCTGCACCCAGTTT
h-*TNF-α*	F:GCCGCATCGCCGTCTCCTAC R:CCTCAGCCCCCTCTGGGGTC
Sus-*GAPDH*	F:ACATGGCCTCCAAGGAGTAAGA R:GATCGAGTTGGGGCTGTGACT
Sus-*CCL2*	F:ATCTTCAAGACCATCGCGGG R:TCAAGGCTTCGGAGTTTGGTT
Sus-*IL8*	F:TGGCAGTTTTCCTGCTTTCT R:CAGTGGGGTCCACTCTCAAT
Sus-*TNF-α*	F:GCCCAAGGACTCAGATCATC R:GGCATTGGCATACCCACTCT
*I10L*	F:ACAGATACGGATTGTAAGGA R:ATCAGCAGTAGTGGCATTA

### Confocal microscopy

Confocal microscopy assay was performed as described previously (44). Briefly, at 24 hours post-transfection, HEK293T cells were fixed with 4% paraformaldehyde for 30 minutes and permeabilized for 20 minutes using 0.1% Triton X-100. Next, the cells were blocked with 1% BSA for 30 minutes and stained with DAPI (catalog no. 36308E; Yeasen) or mouse monoclonal antibody (MAb) against Flag (catalog no. 66008-4-Ig; Proteintech), rabbit MAb against IKK*β* (catalog no. 8943S; Cell Signaling Technology), NF-*κ*B p65 (catalog no. 8242S; Cell Signaling Technology), rabbit polyclonal antibodies (PAbs) against NF-*κ*B1 (catalog no. A14754; ABclonal), or IKK*γ* (catalog no. A12536; ABclonal). Lastly, the cells were imaged using a Zeiss confocal microscope with a 63× oil objective.

### Co-IP and immunoblotting analysis

The cells transfected with plasmids expressing indicated proteins were lysed with M2 lysis buffer (45) [20 mM Tris-HCl (pH 7.5), 0.5% NP-40, 10 mM NaCl, 3 mM EDTA, and 3 mM EGTA] containing protease inhibitors, and sonicated for 2 minutes. The lysates were centrifuged at 13,000× *g* for 10 minutes at 4°C. The supernatants were immunoprecipitated with the mouse anti-Flag MAb, rabbit anti-HA (catalog no. 66006-2-Ig; Proteintech), anti-Myc (catalog no. 60003-2-Ig; Proteintech), anti-IKK*β*, or anti-NF-*κ*B p65 MAb, or rabbit anti-NF-*κ*B1 or anti-IKK*γ* PAb or anti-Flag M2 Magnetic Beads (catalog no. M8823, Sigma). The beads were then washed three times with cold M2 lysis buffer. Bound proteins were separated through SDS-PAGE, followed by immunoblotting analysis.

### GST pulldown assay

To prepare *Escherichia coli* for pI10L expression, the recombinant plasmid pGEX-6p-1-pI10L was transformed into *E. coli* (BL21) cells. The cells in the logarithmic phase were then incubated with IPTG (0.8 mmol/L) for 12 hours at 20°C. After centrifugation, enriched bacterial cultures were resuspended and lysed using a high-pressure homogeneous sterilizer. The supernatants containing recombinant GST or GST-pI10L protein were purified using ChromoTek GST-Trap Agarose beads (catalog no. sta; Proteintech). GST and GST-pI10L were incubated with cell lysates containing the ectopically expressed IKK*β*, NEMO, p65, or p50 at 4°C for 12 hours. Subsequently, the bound proteins were separated by SDS-PAGE, followed by immunoblotting analysis.

### Kinase assay

The flag-tagged pI10L, IKK*β*, p65, or I*κ*B*α* were ectopically expressed and collected for immunoprecipitation assay with anti-Flag M2 magnetic beads (catalog no. M8823; Sigma). Each enriched protein was dissolved in kinase reaction buffer (46) [25 mM Tris-HCl (pH 7.5), 0.01% Triton X-100, 10 mM MgCl_2_, 0.5 mM Na_3_VO_4_, 2.5 mM DTT, 0.5 mM EGTA], with or without 100 *µ*M ATP. Equal amounts of substrates I*κ*B*α* or p65, with or without IKK*β* and pI10L, were subjected to the kinase assay in the reaction buffer and incubated at 37°C for 3 hours. The reaction was stopped with SDS loading buffer, and the bound proteins were separated by SDS-PAGE, followed by immunoblotting analysis with mouse anti-Flag MAb, rabbit anti-phospho-NF-*κ*B p65^Ser536^ (catalog no. 3033S; Cell Signaling Technology) or anti-phospho-I*κ*B*α* ^Ser32^ (catalog no. 2859S; Cell Signaling Technology) MAb.

### Subcellular fractionation

To separate the nuclear fraction (Nuc) and cytoplasmic fraction (Cyt), PK-15 cell lines or PAMs under the treatment of TNF-*α* were washed three times with PBS and once in hypotonic buffer (10 mM Tris-HCl pH 7.4, 10 mM KCl, 1.5 mM MgCl_2_) (47) supplemented with the protease inhibitor PMSF, resuspended in hypotonic buffer, and lysed by leaching homogenization. The lysates were centrifuged at 4°C for 10 minutes at 500× *g* to obtain Nuc pellets, and the supernatants were collected as Cyt supernatant. Nuc pellets were lysed in RIPA buffer with an equal volume of Cyt supernatant, and subjected to western blot analysis with mouse anti-Flag or anti-*β*-actin (catalog no. CL594-66009; Proteintech) MAb, rabbit anti-lamin B1 PAb (catalog no. 12987-1-AP; Proteintech), or rabbit anti-NF-*κ*B p65 MAb.

### Generation and identification of the I10L-deleted ASFV mutant

The recombinant transfer vector pOK12-p72EGFP-ΔI10L, which harbors genomic sequences flanking the targeted gene mapping approximately 1.2 kb upstream and downstream homologous arms, and a reporter gene cassette containing the EGFP reporter under the control of the ASFV *p72* gene promoter, was constructed. The left and right arms flanking the target gene were located in the ASFV_WT_ genome at positions 180919-182119 and 182632-183832, respectively. The nucleotides in the genome at positions 182120–182632 were replaced with an expression cassette containing the EGFP reporter. Briefly, the left and right arms were amplified using PCR and assembled to contain an EGFP reporter harboring restriction enzyme sites at both termini using overlapping PCR. The cassette was cloned into the linearized pOK12 vector to generate the recombinant transfer vector pOK12-p72EGFP-ΔI10L using the Vazyme ClonExpress II one step cloning kit (Vazyme Biotech Co., Ltd., China).

The *I10L*-deleted ASFV mutant ASFV_ΔI10L_ was generated by homologous recombination between the ASFV_WT_ genome and the recombination transfer vector, using infection and transfection procedures in PAMs. This construct generated the expected deletion of the *I10L* gene. The virus resulting from homologous recombination was purified using successive limiting dilutions of PAMs. The purified ASFV_ΔI10L_ was amplified in PAMs to produce a viral stock. To ensure the absence of the desired deletion in each recombinant genome, viral DNA was extracted from ASFV_ΔI10L_-infected PAMs and identified by PCR using specific primers targeting these genes. The primers used in this study are listed in Table 2.

### Virus growth curve

PAM monolayers were prepared in 24-well plates and infected with ASFV_ΔI10L_ or ASFV_WT_ at an MOI of 0.01. After 1 hour of adsorption, the cells were rinsed twice with PBS. The monolayers were incubated in the medium for 2, 12, 24, 48, 72, 96, or 120 hours. At different time points, the ASFV-infected cultures were stored at −70°C. Subsequently, the thawed lysates were used to determine the viral titers in HAD_50_/mL in PAMs.

### Hemadsorption assay

Approximately 5 × 10^4^ PAMs were seeded in 96-well plates and infected with ASFV_ΔI10L_ or ASFV_WT_ for 48 hours. The cells were then incubated with 5 × 10^5^ porcine red blood cells diluted in PBS, and hemadsorption was observed on the fifth day.

### Statistics analysis

GraphPad Prism and SPSS Statistics were used for statistical analysis. Quantitative data in histograms are shown as means ± SD. The data were analyzed using the Log-rank (Mantel-Cox) test or the unpaired Student’s *t* test. The number of asterisks represents the degree of significance with respect to *P* values. Statistical significance was set at *P* < 0.05. *P* values are indicated by asterisks in the figures as follows: *P < 0.05; **P < 0.01; ***P < 0.001.
